# Association between triglyceride-glucose index and worsening renal function in the elderly

**DOI:** 10.3389/fnut.2022.951564

**Published:** 2022-11-24

**Authors:** Li Lei, Hongbin Liang, Yali Qu, Qianhong Zhong, Qiuxia Zhang, Lei Dai, Junyan Lu, Min Xiao, Zhimeng Zhao, Fengyun Zhou, Yun Li, Guifang Hu, Jiancheng Xiu, Xinlu Zhang

**Affiliations:** ^1^Department of Cardiology, Nanfang Hospital, Southern Medical University, Guangzhou, China; ^2^Department of Epidemiology, School of Public Health, Southern Medical University, Guangzhou, China; ^3^Department of Respiratory Medicine, The Fourth People's Hospital of Foshan (Tuberculosis Control Center of Foshan), Foshan, China; ^4^Department of Cardiology, Nanfang Hospital Zengcheng Branch, Guangzhou, China; ^5^Department of Public Health Management, Zengcheng Xintang Hospital, Guangzhou, China

**Keywords:** triglyceride-glucose index, insulin resistance, worsening renal function, elderly, cohort study, insulin resistance

## Abstract

**Background:**

Triglyceride-glucose (TyG) index is a simple marker of insulin resistance. However, insufficient data is available on whether the TyG index is associated with worsening renal function (WRF) in the elderly. Therefore, this study was designed to explore the association between the TyG index and WRF based on a community elderly cohort.

**Methods:**

In this study, 7,822 elderly (aged ≥ 65 years) adults from southern China were enrolled and divided into four groups according to the TyG index quartiles. The primary endpoint was incident chronic kidney disease (CKD), defined as incident estimated glomerular filtration rate (eGFR) < 60 mL/min/1.73 m^2^. Additional endpoints included a decline in eGFR of 30% and 40% during the follow-up period.

**Results:**

During the median 2.04 year follow-up period, 1,541 (19.7%) participants developed CKD. After adjusting for confounding factors, multivariable Cox regression models revealed significant associations between TyG index and incident CKD (HR per SD increase, 1.21; 95% CI: 1.14–1.29), a decline in eGFR of 30% (HR per SD increase, 1.38; 95% CI: 1.26–1.50), and decline in eGFR of 40% (HR per SD increase, 1.42; 95% CI: 1.24–1.63). Furthermore, compared with those in Q1, participants in Q4 demonstrated a higher risk of developing CKD (HR, 1.59; 95% CI: 1.35–1.88). These positive associations remained consistent across different subgroup populations.

**Conclusion:**

Our study suggests a positive and independent association between the TyG index and WRF in the elderly.

## Introduction

Worsening renal function, with a global prevalence of 9.1% in 2017, is an important public health issue associated with cardiovascular diseases and various adverse events (1–3). Globally, premature mortality from worsening renal function has been increasing, and the years of life lost caused by chronic kidney disease (CKD) have increased by 53% since 1990 (1, 4). It is also estimated that the fifth leading cause of global death will be CKD by 2040 (5).

Evidence showed that insulin resistance (IR) is among the most essential risk factors for the progressive decline in renal function (6–8). However, the current gold standard for the assessment of IR, hyperinsulinemic-euglycemic clamp, is too complex for clinical routine, especially in primary care centers (9). Therefore, some surrogate markers of IR emerged.

Triglyceride-glucose (TyG) index, calculated with fasting triglycerides and glucose, is a simple but reliable marker indicating IR (10). Previous studies have proven that the TyG index has a significant correlation with IR assessed by the Homeostatic Model Assessment for Insulin Resistance (HOMA-IR) and the gold standard, hyperinsulinemic-euglycemic clamp (11, 12).

Recently, some studies have reported a positive association between the TyG index and the risk of arterial stiffness, atherosclerosis, and microvascular damage (13–15). Furthermore, several cross-sectional evidence demonstrated that the TyG index is associated with a higher prevalence of CKD, whereas evidence from the longitudinal studies remains insufficient, especially for the elderly (15, 16). Therefore, our study aimed to explore the association between the TyG index and worsening renal function in the elderly based on a retrospective cohort from southern China.

## Materials and methods

### Study population

Data from this retrospective cohort study was derived from the Chinese National Basic Public Health Service project, which includes free general health check-ups annually for the elderly or those with chronic diseases (17). Generally, 9,846 elderly residents (aged ≥ 65 years) attending the annual health check-up at least two times were enrolled, which included 6,554 residents from 37 communities of Guangzhou who attended between January 2017 and July 2021 and 3,292 residents from communities of Foshan who attended between January 2017 and December 2019. In the current analysis, residents with baseline CKD (*n* = 1,972) and insufficient baseline data to calculate the TyG index (*n* = 52) were excluded. The final analysis was conducted based on 7,822 participants (Supplementary Figure S1). The Ethics Committee of the Nanfang Hospital reviewed and approved this study (NFEC-2021-083).

### Study endpoints and major definitions

The primary endpoint of this study was incident CKD, defined as the incident-estimated glomerular filtration rate (eGFR) < 60 mL/min/1.73 m^2^ during the follow-up period (18). Additional endpoints included a decline in eGFR of 30% and 40% during the follow-up period (18, 19). The Chronic Kidney Disease Epidemiology Collaboration (CKD-EPI) equation was used to calculate eGFR (20). The TyG index was calculated as ln[fasting triglycerides (mg/dL) ^*^ fasting glucose (mg/dL)/2] (10). Hypertension was diagnosed if systolic blood pressure (SBP) ≥ 140 mmHg or diastolic blood pressure (DBP) ≥ 90 mmHg or taking any antihypertensive medications (18). Diabetes was diagnosed if fasting glucose ≥ 7 mmol/L or undergoing glucose-lowering therapy or having a history of diabetes diagnosed by physicians (21).

### Data collection

The collected data included demographic characteristics, anthropometric measurements, laboratory assays, smoking status, alcohol use habits, exercise frequency, and medications. All relevant data were derived from the personal electronic health records from the regional chronic disease management platform.

For each individual, his/her first annual health check-up record within the study time window was taken as the baseline. After enrollment, all participants were asked to attend the annual health check-up during each of the following years, and similar check-up contents were provided and results were recorded.

### Statistical analysis

Continuous variables with normal distribution were expressed as mean ± standard deviation while those with non-normal distribution were expressed as median and interquartile range. Categorical variables were demonstrated as percentages. Included subjects were further divided into four groups based on the quartiles of the TyG index, and differences between groups were compared through analysis of variance (ANOVA) or the Kruskal–Wallis test for continuous variables and the chi-square test for categorical variables.

Univariable and multivariable Cox regression models were built to explore the association between the TyG index and worsening renal function. The first multivariable model was adjusted for well-accepted CKD risk factors, including age, sex, diabetes, SBP, DBP, BMI, waist circumference, baseline eGFR, and total cholesterol. The second one was further adjusted for exercising daily, drinking daily, and current smoking status in addition to terms in model 1. In these Cox models, the TyG index was entered as a categorical variable (Q1 as reference) and a continuous variable, respectively. Adjusted restricted cubic spline (RCS) with 4 knots and Cox regression model where TyG index quartile was treated as an ordinal variable were performed to evaluate the linear trend. Participants who lost to follow-up or died were censored at the date of the last examination. For sensitivity analysis, we conducted a multivariable competing risk model among residents from Guangzhou who had follow-up for mortality. Also, we have conducted further analysis stratified by age (65–74 years or ≥ 75 years), sex, hypertension, diabetes, BMI (<25 kg/m^2^ and BMI ≥ 25 kg/m^2^), baseline kidney functions (eGFR 60–90 mL/min/1.73 m^2^ and eGFR > 90 mL/min/1.73 m^2^), triglycerides (< 1.7 mmol/L and ≥ 1.7 mmol/L), and fasting glucose (< 7 mmol/L and ≥7 mmol/L) to explore the differences among these subgroups. Statistical significance was defined as *P*-value < 0.05. All analyses were performed using R software (version 4.2.0; R Foundation for Statistical Computing, Vienna, Austria).

## Results

### Baseline characteristics

The baseline characteristics and missing values of the included subjects are demonstrated in Supplementary Table S1. Baseline characteristics stratified by the TyG index quartiles are displayed in Table 1. In general, the mean age of all included subjects was 70.84 ± 5.27 years, and 59.4% were women. The median and interquartile of the baseline TyG index was 8.65 [8.26; 9.08]. Compared with those with lower TyG index, residents with higher TyG index tended to have higher blood pressure, waist circumference, BMI, fasting glucose, total cholesterol, and triglyceride. Also, participants with higher TyG index had a higher proportion of hypertensive treatment and glucose-lowering treatment. Significant differences between groups were also detected in age, sex, heart rate, smoking status, alcohol use habit, and exercise frequency.

**Table 1:** Baseline characteristics of study participants stratified by triglyceride-glucose index quartiles.

	**Q1**	**Q2**	**Q3**	**Q4**	***P*** **-value**
	**(≤ 8.26)**	**(8.27–8.65)**	**(8.66–9.08)**	**(≥ 9.09)**	
N	1955	1956	1955	1956	
Age, years	70.90 ± 5.47	71.11 ± 5.43	70.84 ± 5.15	70.51 ± 4.98	0.005
Male, *n* (%)	967 (49.5)	789 (40.3)	687 (35.1)	730 (37.3)	<0.001
Heart rate, bpm	74.95 ± 11.69	75.74 ± 11.51	76.66 ± 11.72	78.72 ± 12.71	<0.001
SBP, mmHg	140.87 ± 19.28	143.27 ± 19.03	145.70 ± 19.19	146.32 ± 19.34	<0.001
DBP, mmHg	79.59 ± 11.26	80.41 ± 11.15	81.84 ± 11.15	82.44 ± 10.83	<0.001
Waist, cm	80.86 ± 9.60	84.21 ± 9.51	86.80 ± 9.41	88.61 ± 8.73	<0.001
BMI, kg/m^2^	22.52 ± 3.45	23.63 ± 3.49	24.51 ± 3.38	25.19 ± 3.34	<0.001
Hypertension, *n* (%)	1097 (56.2)	1200 (61.4)	1282 (65.6)	1296 (66.5)	<0.001
Diabetes, *n* (%)	122 (6.2)	241 (12.3)	361 (18.5)	841 (43.0)	<0.001
Current smoker, *n* (%)	265 (13.6)	234 (12.0)	192 (9.8)	211 (10.8)	0.002
Drinking Daily, *n* (%)	87 (4.5)	69 (3.5)	54 (2.8)	65 (3.3)	0.036
Exercising Daily, *n* (%)	1134 (58.0)	1159 (59.3)	1250 (63.9)	1238 (63.3)	<0.001
Fasting glucose, mmol/L	4.51 [4.08, 4.95]	4.84 [4.36, 5.37]	5.03 [4.53, 5.68]	5.89 [5.02, 7.30]	<0.001
Baseline eGFR, mL/min/1.73 m^2^	83.34 [73.34, 90.14]	81.67 [71.73, 89.46]	82.09 [72.54, 89.96]	82.15 [71.62, 89.79]	0.013
Total cholesterol, mmol/L	5.07 [4.47, 5.78]	5.38 [4.70, 6.11]	5.58 [4.89, 6.34]	5.67 [4.90, 6.54]	<0.001
Triglyceride, mmol/L	0.82 [0.69, 0.96]	1.22 [1.07, 1.38]	1.71 [1.50, 1.97]	2.67 [2.14, 3.47]	<0.001
Hypertensive treatment, *n* (%)	323 (23.8)	370 (30.4)	394 (34.2)	454 (42.4)	<0.001
Glucose-lowering treatment, *n* (%)	46 (3.3)	87 (6.9)	118 (9.9)	282 (25.5)	<0.001
TyG index	7.96 ± 0.24	8.46 ± 0.11	8.85 ± 0.12	9.56 ± 0.44	<0.001

SBP, systolic blood pressure; DBP, diastolic blood pressure; BMI, body mass index; eGFR: estimated glomerular filtration rate; TyG index, triglyceride-glucose index.

### Association between TyG index and worsening renal function

During the median follow-up period of 2.04 [1.32; 2.94] years, 1,541 of 7,822 (19.7%) participants from Guangzhou and Foshan developed CKD, and 8 of 5,418 (0.15%) residents from Guangzhou died. Participants who developed CKD tended to have higher TyG index compared with those who did not (8.80 ± 0.66 vs. 8.68 ± 0.63, *P* < 0.001).

**Figure 1:**
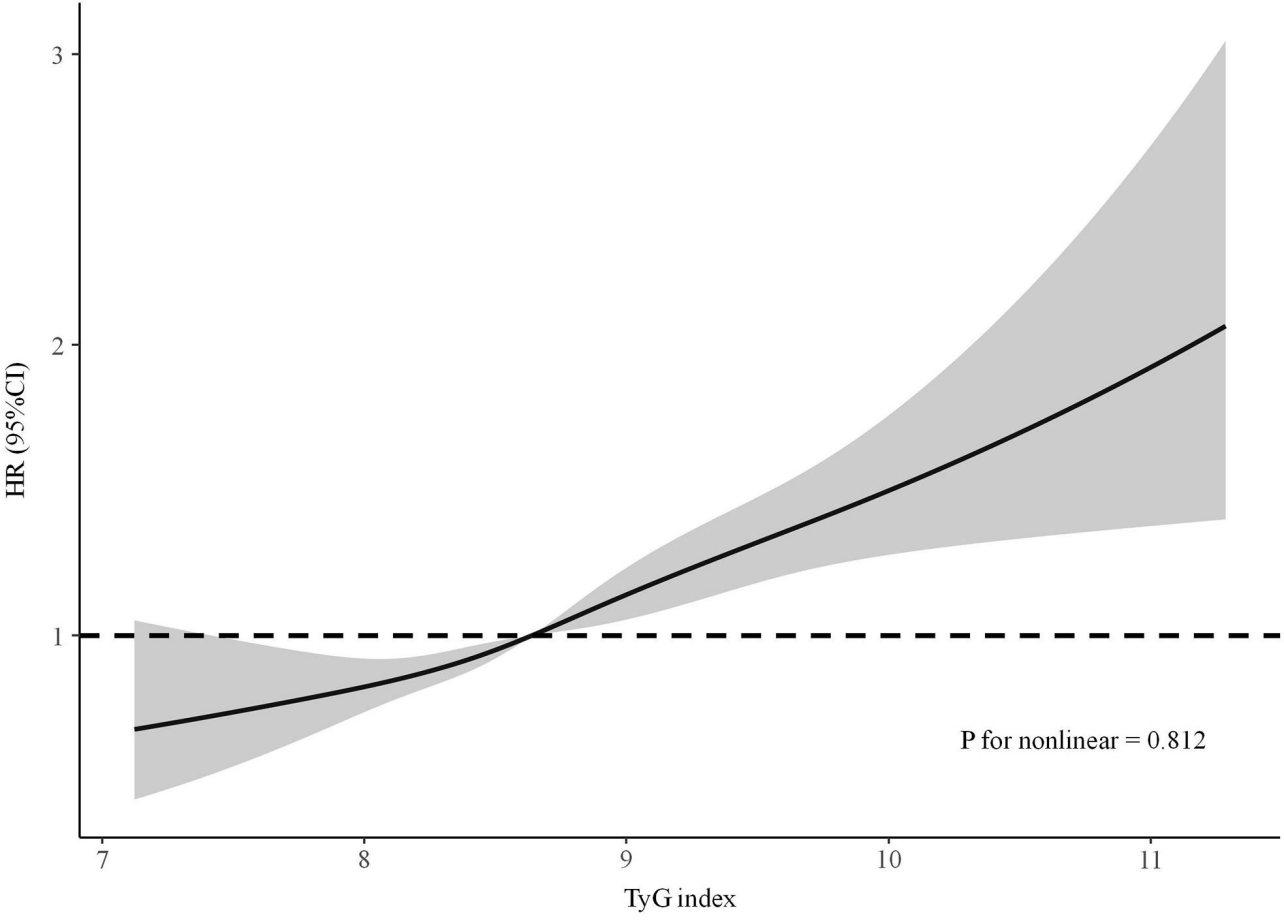
The adjusted restricted cubic spline of the association between the triglyceride–glucose index and incident chronic kidney disease. Adjusted for age, sex, diabetes mellitus, systolic blood pressure, diastolic blood pressure, BMI, waist circumference, baseline eGFR, total cholesterol, exercising daily, drinking daily, and currently smoking.

The results of the Cox regression models are shown in Table 2. In the crude model, the TyG index was significantly associated with incident CKD. After adjusting for the well-accepted CKD risk factors, a significant correlation between elevated TyG index and increased risk of incident CKD was still observed (HR per SD increase, 1.21, 95% CI: 1.14–1.28, *P* < 0.001). Further adjustment for exercising daily, drinking daily, and currently smoking did not alter this positive relationship. In addition, compared with those in the lowest TyG index quartile, participants in the highest quartile tended to have a higher risk of developing CKD even after fully adjusting for confounding factors (HR, 1.59; 95% CI: 1.35–1.88, *P* < 0.001). Linear trend analysis revealed that the risk of incident CKD increased gradually along with the increasing TyG index quartiles with or without adjustment (all P for trend <0.001). Adjusted RCS also revealed a linear association between the TyG index and incident CKD (Figure 1). In the multivariable competing risk model, the TyG index remained significantly associated with incident CKD (HR per SD increase, 1.18, 95% CI: 1.10–1.27, *P* < 0.001).

**Table 2:** Association between triglyceride-glucose index and incident chronic kidney disease.

		**Crude model**		**Adjusted model 1** **^†^,**		**Adjusted model 2** **^‡^**	
	**Event (%)**	**HR (95% CI)**	***P*** **–value**	**HR (95% CI)**	***P*** **–value**	**HR (95% CI)**	***P*** **–value**
**Incident chronic kidney disease**
TyG index per SD increase	1541 (19.7)	1.23 (1.17–1.29)	<0.001	1.21 (1.14–1.28)	<0.001	1.21 (1.14–1.29)	<0.001
**Quartiles**
Q1	312 (16.0)	Reference		Reference		Reference	
Q2	379 (19.4)	1.28 (1.10–1.48)	0.001	1.12 (0.96–1.31)	0.143	1.13 (0.97–1.31)	0.129
Q3	396 (20.3)	1.37 (1.18–1.59)	<0.001	1.31 (1.12–1.53)	<0.001	1.32 (1.13–1.54)	<0.001
Q4	454 (23.2)	1.74 (1.51–2.01)	<0.001	1.59 (1.35–1.87)	<0.001	1.59 (1.35–1.88)	<0.001
P for trend		<0.001		<0.001		<0.001	

TyG index, triglyceride–glucose index.

† Adjusted for age, sex, diabetes mellitus, systolic blood pressure, diastolic blood pressure, BMI, waist circumference, baseline eGFR, and total cholesterol.

‡ Adjusted for all the variables in model 1, plus exercising daily, drinking daily, and currently smoking.

As for additional endpoints, after fully adjusting for well-accepted CKD risk factors and lifestyles, the TyG index (per SD increase) was significantly associated with a decline in eGFR of 30% (HR, 1.38; 95% CI: 1.26–1.50, *P* < 0.001) and 40% (HR, 1.42; 95% CI: 1.24–1.63, *P* < 0.001) (Supplementary Figures S2, S3).

### Subgroup analysis

**Figure 2:**
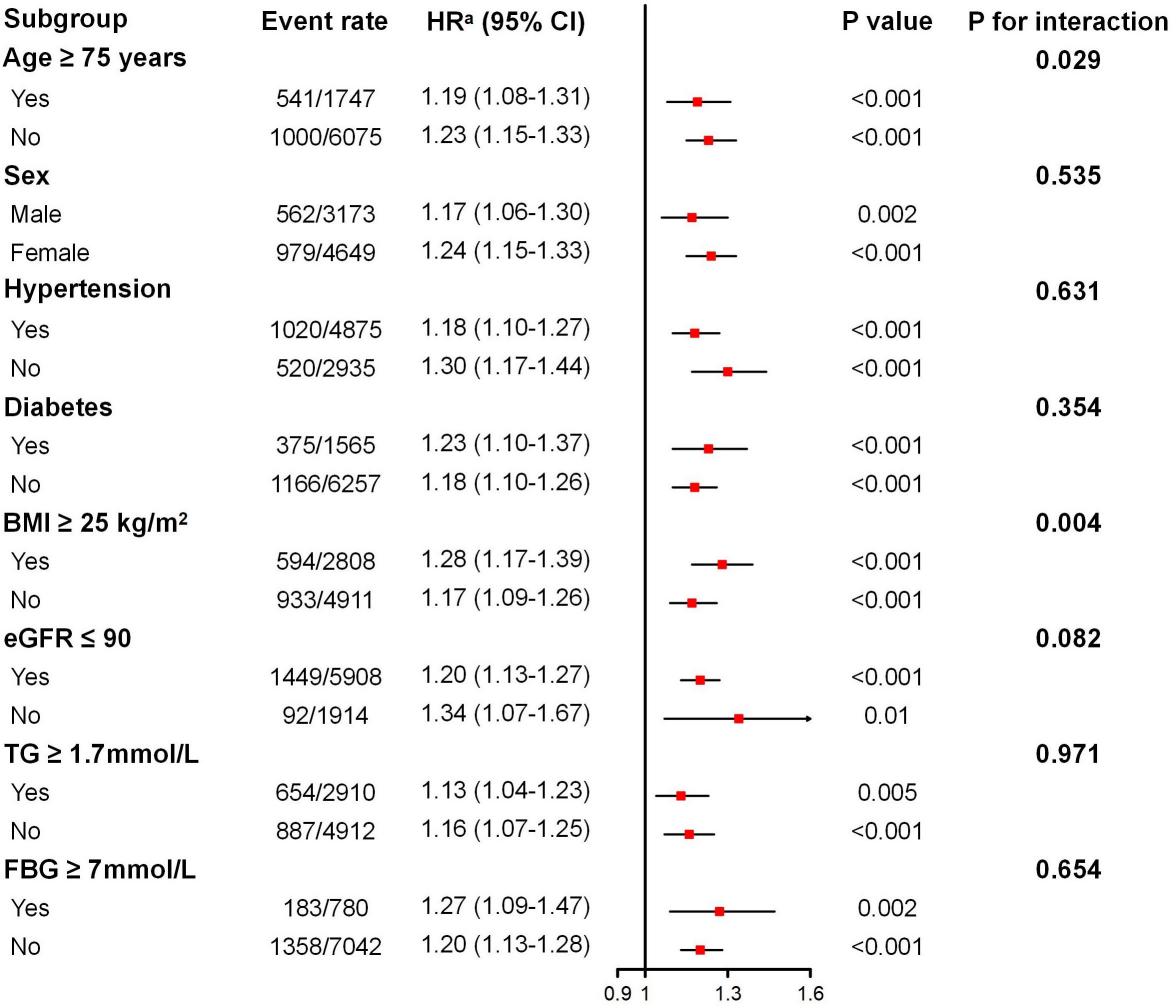
Subgroup analysis of the association between the triglyceride–glucose index and incident chronic kidney disease. BMI, body mass index; eGFR, estimated glomerular filtration rate; TG, triglyceride; FBG, fasting blood glucose. Adjusted for age, sex, systolic blood pressure, diastolic blood pressure, BMI, waist circumference, baseline eGFR, total cholesterol, exercising daily, drinking daily, and currently smoking for diabetes and fasting blood glucose subgroups. Adjusted for age, sex, diabetes mellitus, systolic blood pressure, diastolic blood pressure, BMI, waist circumference, baseline eGFR, total cholesterol, exercising daily, drinking daily, and currently smoking for the other subgroups. ^a^HR given per SD increase.

The associations between the TyG index (per SD increase) and incident CKD in various subgroups are shown in Figure 2. After adjusting for confounding factors, the positive association between TyG index and incident CKD remains consistent across different ages, sexes, BMIs, baseline kidney functions, triglycerides, fasting glucose, and patients with or without hypertension or diabetes, whereas elevated TyG index seemed to be more dangerous for those aged 65 to 74 years (P for interaction = 0.029). For additional endpoints, subgroup analysis revealed a consistently positive association between the TyG index and a decline in eGFR of 30% and 40% in patients with different baselines, whereas a non-significant association was observed for a decline in eGFR of 40% in people with fasting glucose ≥ 7 mmol/L and triglycerides ≥ 1.7 mmol/L (Supplementary Figures S2, S3).

## Discussion

Generally, as a retrospective cohort study with a relatively large sample size of older people, our study reported a positive and independent association between the TyG index and worsening renal function among community-dwelling elderly adults. This significant association between the TyG index and worsening renal function remained consistent across different ages, sexes, BMIs, baseline kidney functions, triglycerides, fasting glucose, and patients with or without diabetes or hypertension. Moreover, an elevated TyG index seemed to be more dangerous for those aged 65 to 74 years.

During a median of 2.04 year follow-up, the incidence of CKD in our cohort was 19.7%, which was higher than previous studies in similar community settings. A prospective community-based study in South Korea enrolled 7,743 middle-aged residents (mean age 52.0 ± 8.8 years), and their reported incidence of CKD was 18.2% during a median follow-up period of 11.5 (1.7–12.5) years (22). Another study in China enrolled 4,042 community residents aged 40 years or older. With follow-up evaluations after a mean of 4.4 years, 355 (8.78%) subjects developed CKD (23). Subjects in our study were much older than those in the above studies (mean age: 70.84 ± 5.27 years). Since age is an important risk factor for renal function deterioration, older participants in our study may be the main reason for higher CKD incidence (18).

A major discovery of the current study was that an elevated TyG index is associated with an increased risk for worsening renal function. IR has been proven among the most essential risk factor for worsening renal function (6–8). Clinically, the gold standard test of IR is too difficult to carry out (9). Therefore, some reliable surrogate markers of IR, like the TyG index, emerged. Recently, several observational studies reported that the TyG index was associated with various chronic diseases, including hypertension and atherosclerotic disease (14, 24). Some cross-sectional studies also reported a potential association between the TyG index and CKD, whereas observations from the longitudinal studies are still insufficient, especially for the elderly. A cross-sectional study enrolled 2,830 community-dwelling elderly individuals in Shanghai and reported a positive association between the TyG index and a higher risk of nephric microvascular damage, which included CKD and microalbuminuria (15). In another cross-sectional study including 1,432 type-2 diabetes patients, a higher TyG index was associated with a higher risk for microalbuminuria (OR, 2.34, 95% CI: 1.74–3.14, *P* < 0.001), as well as eGFR <60 mL/min/1.73 m^2^ (OR, 1.70, 95% CI: 1.10–2.63, *P* = 0.018) (16). In our retrospective cohort study including 7,822 community-dwelling elderly adults, we observed that an elevated TyG index is associated with an increased risk for worsening renal function, which is similar to the previous observations (25). In a historical cohort study of 11,712 general participants from Japan, researchers found that the TyG index was significantly associated with risks for incident CKD (defined as eGFR < 60 mL/min/1.73 m^2^) in both men and women (HR for men, 1.32, 95% CI: 1.02–1.70, *P* = 0.036; HR for women, 1.50, 95% CI: 1.05–2.13, *P* = 0.024) (26). Moreover, in another population-based cohort study of 176,420 individuals from Austria, researchers discovered that the TyG index seemed to be correlated with developing end-stage kidney disease (HR per SD increase, 1.68, 95% CI: 1.56–1.82) (27).

Another finding of our study was that elevated TyG index is not only associated with incident CKD but also eGFR decline. GFR decline, which is associated with a higher risk for cardiovascular events (28, 29), has been recommended as a surrogate endpoint for clinical trials in CKD. Both 30 and 40% are the well-accepted thresholds to define clinically significant eGFR decline (19). In the current study, we found that elevated TyG index is associated with an increased risk for decline in eGFR of 30% and 40% after adjusting for confounding factors, including age, sex, blood pressure, BMI, baseline kidney function, and so on. Furthermore, across different ages, sexes, BMIs, baseline kidney functions, triglycerides, fasting glucose, and patients with or without diabetes or hypertension, this positive association between TyG index and worsening renal function, including incident CKD and decline in eGFR of 30% and 40%, remained consistent and unchanged. Although a non-significant association was observed for a decline in eGFR of 40% in some subgroups, the positive trend remains. And we think that this phenomenon may be due to the limited cases of endpoint in these subgroups. In general, these findings further strengthened our results and emphasized the need for general practitioners to pay more attention to subjects with elevated TyG index.

The mechanism underlying the association between the TyG index and worsening renal function remains uncertain. Generally, IR plays an important role in metabolic disease-induced CKD by causing hyperglycemia and later low-grade inflammation and fibrosis. When IR occurs, adipocytes have difficulty using glucose, which then leads to abnormality in lipid profiles and fasting glucose (6, 8). And this may be the potential reason to explain the significant association between the TyG index, calculated with fasting triglycerides and glucose, and worsening renal function. Still, further explorations are needed.

The strengths of the current study include the relatively large community-based sample from two metropolises of southern China and the discovery of a positive relationship between TyG index and worsening renal function among the elderly. Indeed, compared with HOMA-IR, the TyG index is currently not a well-accepted alternative tool for defining IR. However, due to the limited resources in primary care in China, fasting insulin, a component to calculate HOMA-IR, is generally unavailable in most primary medical institutions. In this context, the advantage of the simplicity of the TyG index is highlighted. In consideration of the results of our study, general practitioners in primary care should pay more attention to those with elevated TyG index, as it is associated with developing not only kidney disease but also various chronic diseases (14, 24).

Our study also had some limitations. First, due to the limited data, some reported risk factors for CKD, such as the albumin-to-creatinine ratio, were not adjusted in our analysis. Second, limited by the annual health check-up items, we are not able to make a direct comparison between the TyG index and HOMA-IR. Third, the frequency of renal function tests in our study was once a year, which can cause an underestimate of the effect of a high TyG index on worsening renal function. A more continuous follow-up of renal function is warranted to settle this problem in future studies.

## Conclusion

In summary, we observed that an elevated TyG index was positively and independently associated with an increased risk for worsening renal function among the elderly. General practitioners in primary care should pay more attention to those with elevated TyG index in future for its clinical significance.

## Data availability statement

Data relevant to this study are available upon reasonable request to the corresponding authors.

## Ethics statement

The studies involving human participants were reviewed and approved by Ethics Committee of Nanfang Hospital, Southern Medical University. The patients/participants provided their written informed consent to participate in this study.

## Author contributions

Conceived and designed the study: LL, HL, GH, JX, and XZ. Acquisition and analysis of data: LL, HL, YQ, QZho, QZha, LD, JL, MX, ZZ, FZ, and YL. Statistical analysis of data and interpretation: LL, HL, YQ, and GH. Wrote the manuscript: LL, HL, YQ, and QZho. Revised the manuscript: JX and XZ. All authors read and approved the final version of the manuscript.

## Funding

This work was supported by the National Key R&D Program of China [Grant Number 2018YFC1312803], National Natural Science Foundation of China (Grant Numbers 81974266 and 81800371), the Clinical Research Program of Nanfang Hospital, Southern Medical University [Grant Number 2021CR007], the Medical Research Foundation of Guangdong Province in 2020 [Grant Number B2020108], A Major Infectious Disease Prevention and Control of the National Science and Technique Major Project [Grant Number 2018ZX10715004], the Guangzhou Health Science and Technology Project [Grant Number 20221A040018], and Overseas Master Program from Guangdong Province Science and Technology Project [Grant Number 109268542061].

## Conflict of interest

The authors declare that the research was conducted in the absence of any commercial or financial relationships that could be construed as a potential conflict of interest.

## Publisher's note

All claims expressed in this article are solely those of the authors and do not necessarily represent those of their affiliated organizations, or those of the publisher, the editors and the reviewers. Any product that may be evaluated in this article, or claim that may be made by its manufacturer, is not guaranteed or endorsed by the publisher.
