# Supplementary material for: Association between triglyceride-glucose index and worsening renal function in the elderly
Source: Front Nutr. 2022 Nov 24;9:951564. doi: 10.3389/fnut.2022.951564 (PMC9730025; doi:10.3389/fnut.2022.951564)
Supplement: Supplementary file 1 [file Table_1.docx]

**Supplementary table 1. Baseline characteristics and missing values of the entire cohort**

|  | Missing value (%) | Overall |
| --- | --- | --- |
| N |  | 7822 |
| Age, years | 0 (0.0) | 70.84 ± 5.27 |
| Male, n (%) | 0 (0.0) | 3173 (40.6) |
| Heart rate, bpm | 30 (0.4) | 76.51 ± 12.00 |
| SBP, mmHg | 22 (0.3) | 144.04 ± 19.33 |
| DBP, mmHg | 27 (0.3) | 81.07 ± 11.15 |
| Waist, cm | 238 (3.0) | 85.10 ± 9.76 |
| BMI, kg/m^2^ | 103 (1.3) | 23.96 ± 3.56 |
| Hypertension, n (%) | 12 (0.2) | 4875 (62.4) |
| Diabetes, n (%) | 0 (0.0) | 1565 (20.0) |
| Current smoker, n (%) | 1 (0.0) | 902 (11.5) |
| Drinking Daily, n (%) | 4 (0.1) | 275 (3.5) |
| Exercising Daily, n (%) | 1 (0.0) | 4781 (61.1) |
| Fasting glucose, mmol/L | 0 (0.0) | 4.95 [4.41, 5.70] |
| Baseline eGFR, mL/min/1.73m^2^ | 0 (0.0) | 82.36 [72.32, 89.90] |
| Total cholesterol, mmol/L | 4 (0.1) | 5.43 [4.71, 6.17] |
| Triglyceride, mmol/L | 0 (0.0) | 1.40 [1.00, 2.04] |
| Hypertensive treatment, n (%) | 3027 (38.7) | 1541 (32.1) |
| Glucose-lowering treatment, n (%) | 2870 (36.7) | 533 (10.8) |
| TyG index | 0 (0.0) | 8.71 ± 0.64 |

Abbreviations: SBP, systolic blood pressure; DBP, diastolic blood pressure; BMI, body mass index; eGFR: estimated glomerular filtration rate; TyG index, triglyceride-glucose index.

**Supplementary figure 1. Flow chart of the study design.**


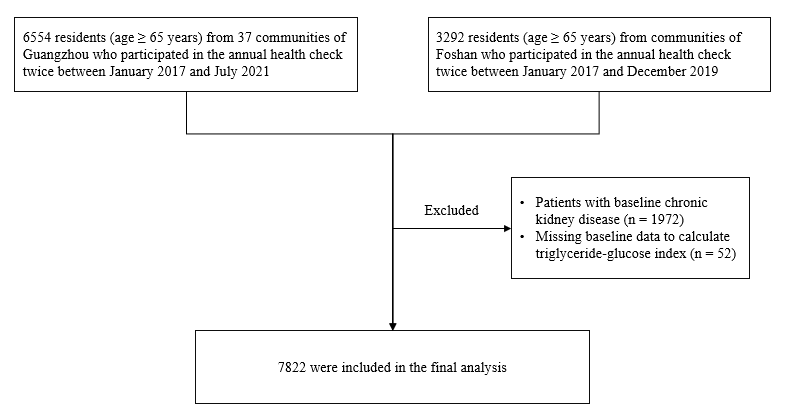


**Supplementary figure 2. Association between triglyceride-glucose index and decline in estimated glomerular filtration rate of 30%.**


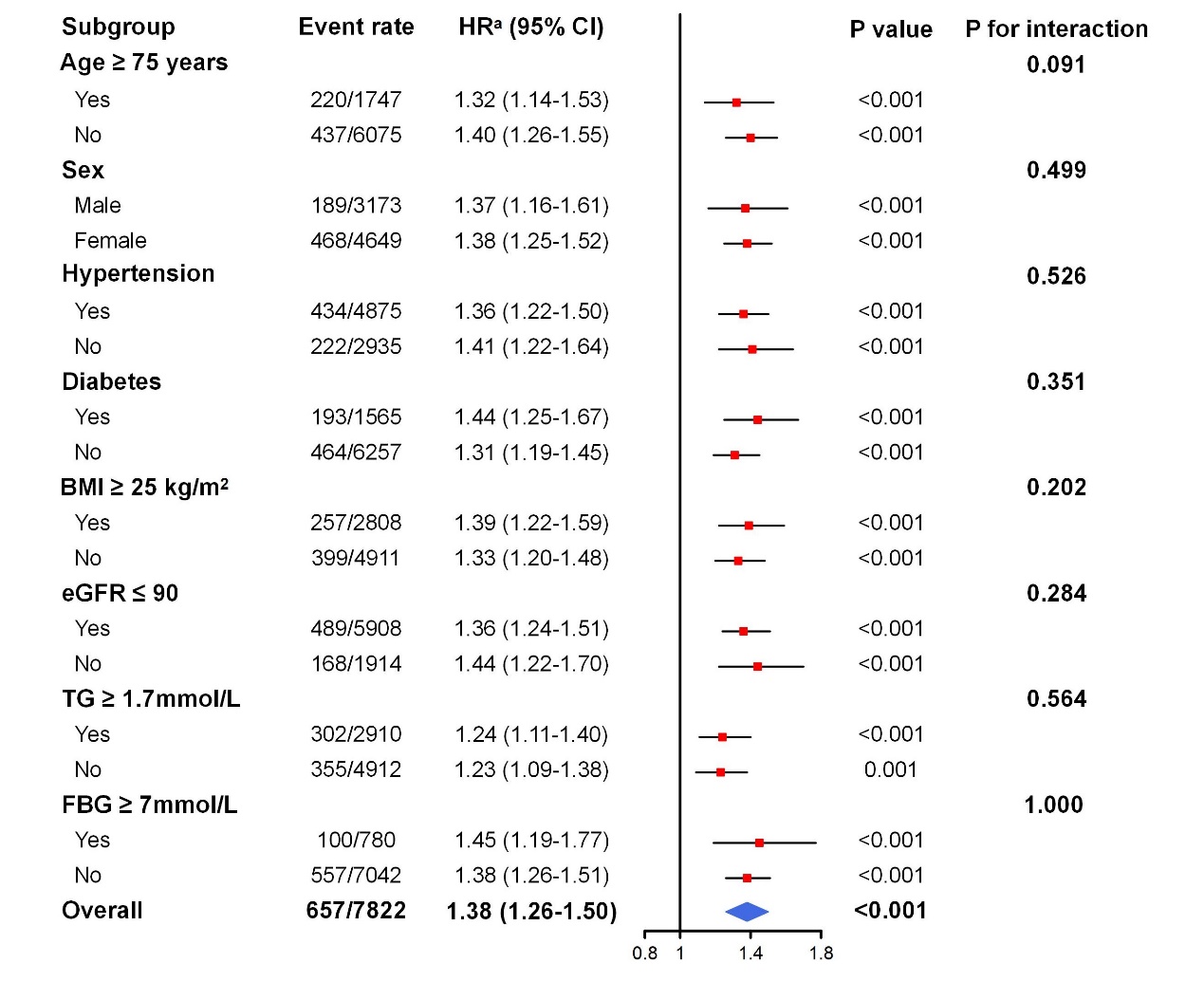


Abbreviations: BMI, body mass index; eGFR, estimated glomerular filtration rate; TG, triglyceride; FBG, fasting blood glucose.

Adjusted for age, sex, systolic blood pressure, diastolic blood pressure, BMI, waist circumference, baseline eGFR, total cholesterol, exercising daily, drinking daily, and currently smoking for the diabetes and fasting blood glucose subgroups.

Adjusted for age, sex, diabetes mellitus, systolic blood pressure, diastolic blood pressure, BMI, waist circumference, baseline eGFR, total cholesterol, exercising daily, drinking daily, and currently smoking for the other subgroups.

^a^HR given per SD increase.

**Supplementary figure 3. Association between triglyceride-glucose index and decline in estimated glomerular filtration rate of 40%.**


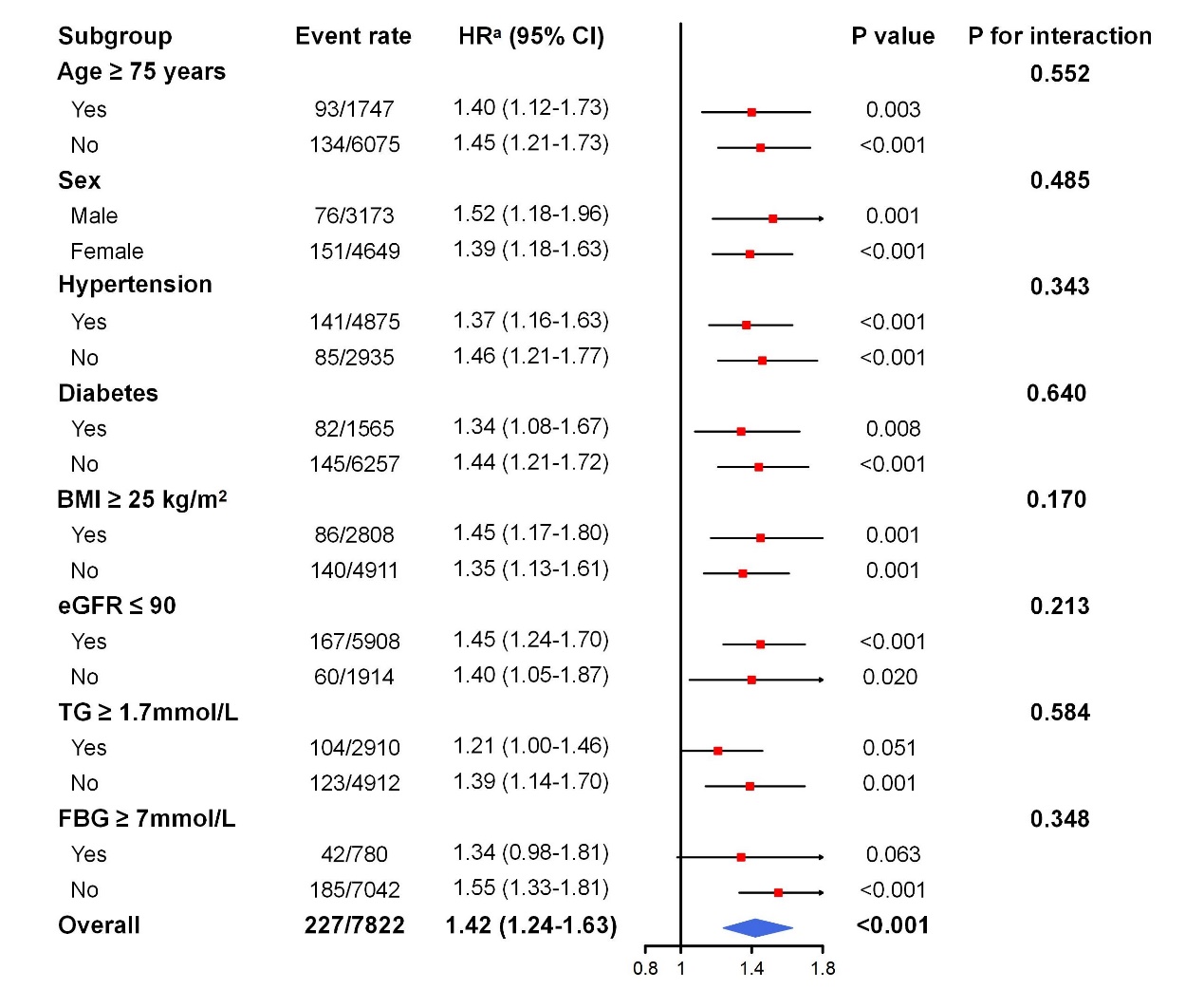


Abbreviations: BMI, body mass index; eGFR, estimated glomerular filtration rate; TG, triglyceride; FBG, fasting blood glucose.

Adjusted for age, sex, systolic blood pressure, diastolic blood pressure, BMI, waist circumference, baseline eGFR, total cholesterol, exercising daily, drinking daily, and currently smoking for the diabetes and fasting blood glucose subgroups.

Adjusted for age, sex, diabetes mellitus, systolic blood pressure, diastolic blood pressure, BMI, waist circumference, baseline eGFR, total cholesterol, exercising daily, drinking daily, and currently smoking for the other subgroups.

^a^HR given per SD increase.
